# Commentary: Manifold Routes to a Nucleus

**DOI:** 10.3389/fmicb.2019.01198

**Published:** 2019-05-31

**Authors:** Christian Jogler, Sandra Wiegand, Damien P. Devos

**Affiliations:** ^1^Department of Microbiology, Radboud University Nijmegen, Nijmegen, Netherlands; ^2^Centro Andaluz de Biología del Desarrollo (CABD)-CSIC, Pablo de Olavide University, Seville, Spain

**Keywords:** nucleus, compartmentation, planctomycetes, nuclear pore, evolution

In the manuscript “Manifold Routes to a Nucleus”, Hendrickson and Poole question the assumption that the evolution of nuclear compartmentalization has been a singular event. They provide interesting observations on jumbophages and newly described archaea. However, their claim that bacteria from the *Planctomycetes* phylum display “nucleus-like compartmentation” is based on an outdated interpretation of the planctomycetal cell plan (Fuerst and Sagulenko, 2011). Various comprehensive reviews on the paradigm shift in the field of planctomycetal research were recently published (Devos, 2014a,b; Rivas-Marin and Devos, 2018; Wiegand et al., 2018) and are summarized in Figure 1.

**Figure 1:**
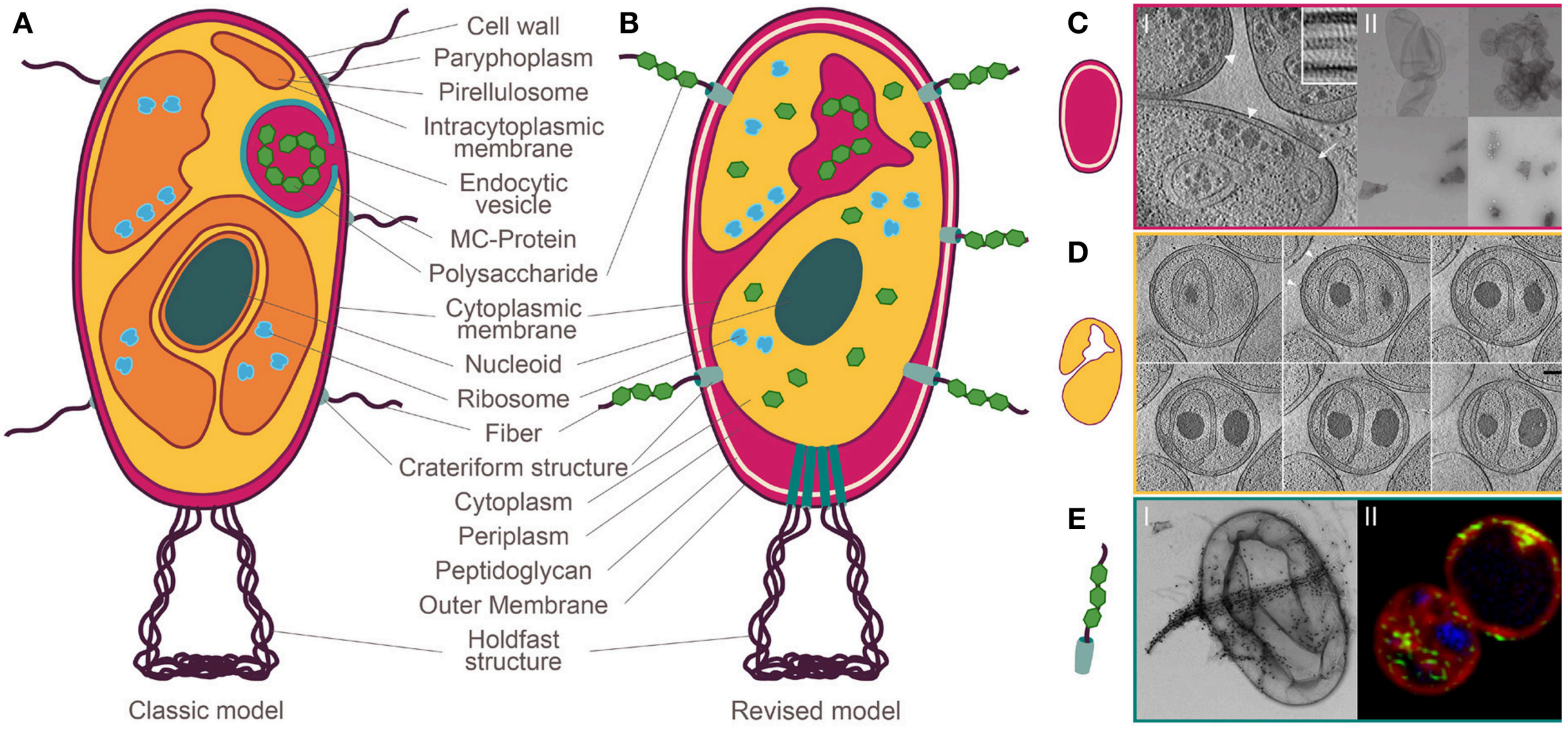
Planctomycetal cell plan before and after the paradigm shift. **(A)** Classic model: compartmentalization into paryphoplasm and pirellulosome with an additional nuclear compartment in *Gemmata obscuriglobus*. A peptidoglycan layer is missing in favor of a proteinaceous cell wall. Complex macromolecules are ingested by endocytosis. **(B)** Revised model: Gram-negative cell plan including an outer membrane, a cell wall formed of peptidoglycan and a cytoplasmic membrane. **(C)** Jeske et al. (2015) I: Cryo-electron tomography (CET) of the planctomycetal cell envelope shows three layers, II: preparation of peptidoglycan saccule (top) that have been destroyed by lysozyme (bottom). **(D)** Boedeker et al. (2017) Invaginations into the cytoplasm by extended periplasmic space. **(E)** Boedeker et al. (2017) I: Crateriform structures give rise to fibers that bind macromolecules such as dextran, II: Green-labeled dextran enters the cell through crateriform structures and is stored in the enlarged periplasmic space. Modified after Wiegand and Jogler (2018).

The concept of a nuclear structure outside the eukaryotes was introduced in 1991, when Fuerst and Webb postulated that the planctomycetal species *Gemmata obscuriglobus* harbors a bacterial “nucleus” (Fuerst and Webb, 1991). At this time, *Planctomycetes* were thought to share similarities with bacteria and eukaryotes alike [Figure 1, for review see (Fuerst and Sagulenko, 2011)]. Besides the suggested nucleoid (Fuerst and Webb, 1991), the cytoplasm of *Planctomycetes* was considered to be compartmentalized by an intracytoplasmic membrane (Lindsay et al., 2001). In contrast to all other Gram-negative bacteria, *Planctomycetes* were believed to lack a peptidoglycan sacculus and their outermost membrane was suggested to be the cytoplasmic one and to be surrounded by a proteinaceous cell wall instead (König et al., 1984; Liesack et al., 1986; Stackebrandt et al., 1986). Given this model of a planctomycetal cell (see Figure 1A), it was not surprising that other typical eukaryotic hallmark-traits such as endocytosis (Fuerst and Sagulenko, 2010; Lonhienne et al., 2010) and the separation of translation and transcription were suggested (Gottshall et al., 2014). Various interpretations were proposed, such as *Planctomycetes* are “beyond the bacterium” (Fuerst and Sagulenko, 2011), or that they represent intermediate steps between pro- and eukaryotes (Devos and Reynaud, 2010).

However, the concept of the planctomycetal cell plane evolved (Figure 1B). Others and ourselves have found that *Planctomycetes* possess a peptidoglycan cell wall (Figure 1C; Jeske et al., 2015; Van Teeseling et al., 2015) and the three-dimensional reconstruction of a *G. obscuriglobus* cell revealed interconnections of all previously suggested compartments, including the nucleus-like structure (Santarella-Mellwig et al., 2010) and an interconnected tubulovesicular network (Acehan et al., 2013). These findings of a continuous cytoplasm with invaginations of the cytoplasmic membrane called for a reinterpretation of the classic cell plan (Devos, 2014a,b; Wiegand et al., 2018). We also investigated members of different phylogenetic branches of *Planctomycetes* in a comprehensive study involving mutagenesis and state-of-the-art microscopic techniques and demonstrated that canonical *Planctomycetes* are a variation of, but not an exception to, the Gram-negative cell plan (Devos, 2014a,b; Boedeker et al., 2017). Most importantly, all investigated planctomycetal species lack compartmentalization by additional membranes (Figure 1D; Boedeker et al., 2017). Consequently, there is no separate nucleus-like compartment in *Planctomycetes*. However, unusual large pores known as crateriform structures are found in the planctomycetal outer membrane (Figure 1E; König et al., 1984; Schlesner and Hirsch, 1984; Fuerst et al., 1991; Ward et al., 2006; Boedeker et al., 2017). Such structures are unique among bacteria and can be found on the surface of all *Planctomycetes* (Wiegand et al., 2018). We believe that these crateriform structures have mistakenly been interpreted as “nuclear pores” in a recent study (Sagulenko et al., 2017). Despite these findings, alternative interpretations are still presented (Sagulenko et al., 2014;Feijoo-Siota et al., 2017).

However, we do acknowledge that one aspect of the potential planctomycetal “compartmentalization” requires further investigation: one branch of the phylum, the one of the anammox bacteria, was shown to contain a separated subcellular compartment, the anammoxosome (Neumann et al., 2014). To obtain energy, ammonium is anaerobically oxidized to dinitrogen gas in this compartment whilst forming toxic hydrazine as intermediate metabolite [for review see (Peeters and van Niftrik, 2018)]. Therefore, it was called the “bacterial mitochondrion” (Jogler, 2014). Specific ladderane lipids were thought to protect the anammox membrane against leakage of hydrazine into the cytosol where it would harm the DNA (Sinninghe Damste et al., 2002). In their study, Hendrickson and Pool argue that this is an example for chemical protection of the DNA by an organelle. However, recently it was shown that ladderane membranes are as permeable to hydrazine as straight-chain lipid bilayers (Moss et al., 2018), representing a problem for the authors' hypothesis.

Regarding the main aspect of their article “Manifold Routes to a Nucleus,” Hendrickson and Poole unfortunately do not mention most of the above discussed articles (Speth et al., 2012; Devos, 2014a,b; Rivas-Marin and Devos, 2018; Wiegand et al., 2018). By acknowledging that “the exact nature of this compartmentation is a matter of ongoing debate” they neglect the fact that the described findings do not question the nature of the compartments but their very existence. This disregard leaves the reader under the impression that there is a bacterial nucleus. However, many of their suggestions suffer incoherence when confronted with the most recent data.

Despite the absence of a nucleus-like structure, the phylum *Planctomycetes* and the extended PVC superphylum is still a relevant model for endomembrane formation—maybe related to DNA protection—and in any case for cell organization and complexity.

## Author Contributions

All authors listed have made a substantial, direct and intellectual contribution to the work, and approved it for publication.

### Conflict of Interest Statement

The authors declare that the research was conducted in the absence of any commercial or financial relationships that could be construed as a potential conflict of interest.
